# Verification of inflammation‐based prognostic marker as a prognostic indicator in hepatocellular carcinoma

**DOI:** 10.1002/ags3.12286

**Published:** 2019-09-20

**Authors:** Masateru Yamamoto, Tsuyoshi Kobayashi, Shintaro Kuroda, Michinori Hamaoka, Sho Okimoto, Naruhiko Honmyo, Megumi Yamaguchi, Hideki Ohdan

**Affiliations:** ^1^ Department of Gastroenterological and Transplant Surgery Graduate School of Biomedical and Health Science Hiroshima University Hiroshima Japan

**Keywords:** C‐reactive protein to albumin ratio, Glasgow prognostic score, hepatocellular carcinoma, lymphocyte to monocyte ratio, neutrophil to lymphocyte ratio

## Abstract

**Aim:**

Although inflammation‐based markers in cancer have been used for prognostic prediction, the most useful marker for hepatocellular carcinoma (HCC) has not been established. We investigated the usefulness of various inflammation‐based markers in HCC patients after hepatectomy.

**Methods:**

A total of 478 patients who underwent initial hepatectomy for HCC from 2009 to 2015 and were diagnosed with pathological HCC were included in this retrospective study. Inflammation‐based markers consisted of the C‐reactive protein to albumin ratio (CAR), Glasgow prognostic score (GPS), neutrophil to lymphocyte ratio, lymphocyte to monocyte ratio, platelet to lymphocyte ratio and prognostic index. Univariate and multivariate analyses for overall survival (OS) and disease‐free survival (DFS) using the Cox proportional hazard model were carried out. Kaplan‐Meier analysis and log‐rank test were used for comparison of OS and DFS. To reduce influences of selection bias and confounders for stratifying CAR, clinicopathological characteristics of patients were balanced by propensity score matching.

**Results:**

Multivariate analysis identified only high CAR (>0.027) as an indicator of poor OS, and high CAR and high GPS (1‐2) as indicators of poor DFS among inflammation‐based markers. After propensity score matching, 124 patients each with low CAR and high CAR were matched. High CAR was correlated with both poor OS and DFS.

**Conclusion:**

C‐reactive protein to albumin ratio was the most valuable prognostic indicator after hepatectomy for HCC among inflammation‐based markers.

## Abstract 2

C‐reactive protein to albumin ratio was the most valuable prognostic indicator after hepatectomy for HCC among inflammation‐based markers.

## Graphical abstract

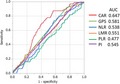

## 1 INTRODUCTION

Hepatocellular carcinoma (HCC) is the fifth most common cancer and the second leading cause of cancer‐related death globally.^1^, ^2^ Liver transplantation is an effective treatment, but the limited number of donor livers prevents widespread use of this oncological therapy.^3^ Surgical resection is hence still the most effective method for the treatment of HCC. However, the high recurrence rate after curative resection indicates that the prognosis of HCC is still insufficient despite recent progress in treatment.^4^ Thus, identification of patients with probable poor prognosis using reliable biomarkers is essential for improving survival outcomes of HCC patients.

Previous studies have shown that various staging systems and serum biomarkers have predicted prognosis using tumor‐related factors.^5^, ^6^, ^7^ There is increasing evidence that inflammation is crucial in the progression of cancer development and the presence of systemic inflammatory response has been shown to be associated with clinical outcomes in several malignancies.^8^, ^9^, ^10^ Recent studies have also proposed markers based on a number of inflammation‐based prognostic indicators of HCC. Inflammation‐based markers include the C‐reactive protein (CRP) to albumin ratio (CAR),^11^ Glasgow prognostic score (GPS),^12^ neutrophil to lymphocyte ratio (NLR),^13^ lymphocyte to monocyte ratio (LMR),^14^ platelet to lymphocyte ratio (PLR)^15^ and prognostic index (PI).^16^

Nevertheless, it remains unclear which inflammation‐based marker more accurately predicts the prognosis after hepatectomy for HCC. In the present study, the value of these inflammation‐based markers as predictive indicators of prognosis was explored in patients with HCC after hepatectomy.

## 2 MATERIALS AND METHODS

### 2.1 Patients

This study was based on a retrospective analysis of 478 HCC patients who underwent hepatectomy at our institution between January 2009 and December 2015. Inclusion criteria were follows: (i) tumor was histologically diagnosed as HCC; (ii) no distant metastasis was detected in the preoperative image; (iii) resection margin was negative; (iv) first hepatectomy for HCC; and (v) no other malignancies. Baseline clinicopathological findings were retrieved and reviewed from the hospital database. Characteristics of patient cohorts are shown in Table 1. This study was the approved by the Institutional Review Board (Provided ID Number: E‐1580) on the basis of the Ethical Guidelines for Clinical Research of the Ministry of Health, Labour and Welfare in Japan.

**Table 1:** Clinicopathological characteristics of enrolled patients with hepatocellular carcinoma

Variable	N = 478
Age (y)	69 (63‐77)
Gender (male/female)	301/177 (63.0/38.0)
BMI (kg/m^2^)	22.9 (20.5‐25.0)
HBV (N/Y)	400/75 (84.2/15.8)
HCV (N/Y)	202/255 (46.5/53.5)
CRP (mg/dL)	0.54 (0.03‐0.20)
Alb (g/dL)	4.03 (3.7‐4.4)
AST (IU/L)	50 (24‐46)
ALT (IU/L)	36 (19‐41)
Plt (×10^4^/mm^3^)	15.3 (10.5‐18.5)
PT (%)	86.2 (79‐95)
T‐Bil (mg/dL)	0.8 (0.4‐1.0)
ICGR15 (%)	15.9 (8.7‐20.0)
AFP (ng/mL)	3222 (5‐50)
DCP (mAU/mL)	3625 (21‐407)
Child‐Pugh (A/B)	439/38 (92.1/7. 9)
Liver damage (A/B/C)	345/122/2 (73.6/26.0/0.4)
Tumor number (1/>1)	311/167 (65.1/34.9)
Tumor size (mm)	40.2 (16.5‐44.0)
Anatomical resection (Y/N)	333/144 (69.9/30.1)
Operation time (min)	326 (249‐386)
Blood loss (mL)	504 (160‐600)
LC (N/Y)	326/122 (72.7/27.3)
MVI (N/Y)	382/96 (79.9/20.1)
Histological grade	
Well/moderately differentiated/poorly differentiated	56/351/51 (12.2/76.7/11.1)
IM (N/Y)	397/51 (88.7/11.3)
CAR (≤0.027/>0.027)	301/177 (62.9/37.1)
GPS (0/1, 2)	376/102 (78.7/21.3)
NLR (≤2.2/>2.2)	293/185 (61.2/38.7)
LMR (≥3.3/<3.3)	364/118 (75.5/24.5)
PLR (≤106/>106)	275/207 (57.1/42.9)
PI (0/1, 2)	435/45 (90.6/9.4)

AFP, alpha‐fetoprotein; Alb, albumin; AST, aspartate aminotransferase; AST, aspartate aminotransferase; BMI, body mass index; CAR, C‐reactive protein to albumin ratio; CRP, C‐reactive protein; DCP, des‐gamma‐carboxyprothrombin; GPS, Glasgow prognostic score; HBV, hepatitis B virus; HCV, hepatitis C virus; ICGR15, indocyanine green retention rate at 15 min; IM, intrahepatic metastasis; LC, liver cirrhosis; LMR, lymphocyte to monocyte ratio; MVI, microvascular invasion; NLR, neutrophil to lymphocyte ratio; PI, prognostic index; PLR, platelet to lymphocyte ratio; Plt, platelet count; PNI, prognostic nutritional index; PT, prothrombin time; T‐Bil, total bilirubin.

Data are presented as mean and interquartile ranges of continuous variables, and as number and percentage for categorized variables.

### 2.2 Treatment and patient follow up

Hepatectomy procedure was determined after evaluating tumor size, number of tumors, tumor location, liver function and patient status. Hepatectomy and liver function were classified according to the General Rules for the Clinical and Pathological Study of Primary Liver Cancer.^17^ After being discharged from the hospital, all patients were screened for tumor recurrence and metastasis using measurements of tumor markers every 3 months, and using abdominal ultrasound, computed tomography and magnetic resonance imaging every 6 months. Duration of follow up was defined as the date of operation to the date of the last follow up before the data were analyzed, or as the date of death.

### 2.3 Definition of inflammation‐based prognostic systems

Prior to the operation, blood samples were collected. CAR was calculated as the patient's serum CRP level (mg/dL) divided by the serum albumin level (g/dL). GPS was calculated as follows: patients with neither an elevated CRP level (>1.0 mg/dL) nor hypoalbuminemia (albumin level <3.5 g/dL) were assigned a GPS of 0, patients with either of these biochemical abnormalities were assigned a GPS of 1, and patients with both elevated CRP and hypoalbuminemia were assigned a GPS of 2. NLR was calculated as the patients’ neutrophil level divided by the lymphocyte level. LMR was calculated as the patients’ lymphocyte level divided by the monocyte level. PLR was calculated as the patients’ platelet level divided by the lymphocyte level. PI was calculated as follows: patients with neither an elevated CRP level (>1.0 mg/dL) nor elevated white blood cell (WBC) count (>11 000/μL) were assigned a PI of 0, patients with either one or the other of these biochemical abnormalities were assigned a PI of 1, patients with both elevated CRP levels and WBC levels were assigned a PI of 2. In addition, prognostic nutritional index (PNI) was assessed; patients with albumin level (mg/dL) × 10 + lymphocyte level × 0.005 ≥45 were assigned a PNI of 0 and albumin level (mg/dL) × 10 + lymphocyte level × 0.005 <45 were assigned a PNI of 1.

### 2.4 Statistical analysis

Continuous variables were expressed as median and range and compared using the Mann‐Whitney *U* test. Categorical variables were expressed as number and percentage and compared using Fisher's exact test. Optimal cut‐off points of CAR, NLR, LMR and PLR for the overall survival (OS) were determined by receiver operating characteristic (ROC) curve analysis. Survival curves were generated using the Kaplan‐Meier method and compared between different groups using the log‐rank test. Multivariate Cox proportional hazards model was used to determine independent risk factors associated with survival. Statistically significant variables in the univariate analysis were entered into the multivariate Cox regression analysis. *P*‐value was considered significant if <.05.

Propensity score matching was used to diminish bias as a result of the different distributions of covariates between the high and low CAR groups. Based on a logistic regression model, propensity scores were analyzed according to baseline characteristics including age, gender, body mass index (BMI), status of hepatitis B and C virus (HBV, HCV), platelet count, prothrombin, aspartate aminotransferase (AST), alanine aminotransferase, indocyanine green retention rate at 15 minutes (ICGR15), alpha‐fetoprotein levels (AFP), des‐gamma‐carboxyprothrombin (DCP), Child‐Pugh grade, liver damage, number of tumors, tumor size, anatomical resection, operation time, blood loss, microvascular invasion (MVI), liver cirrhosis, histological grade, and intrahepatic metastasis (IM). One‐to‐one matching was carried out using a 0.20 caliper.

## 3 RESULTS

**Figure 1:**
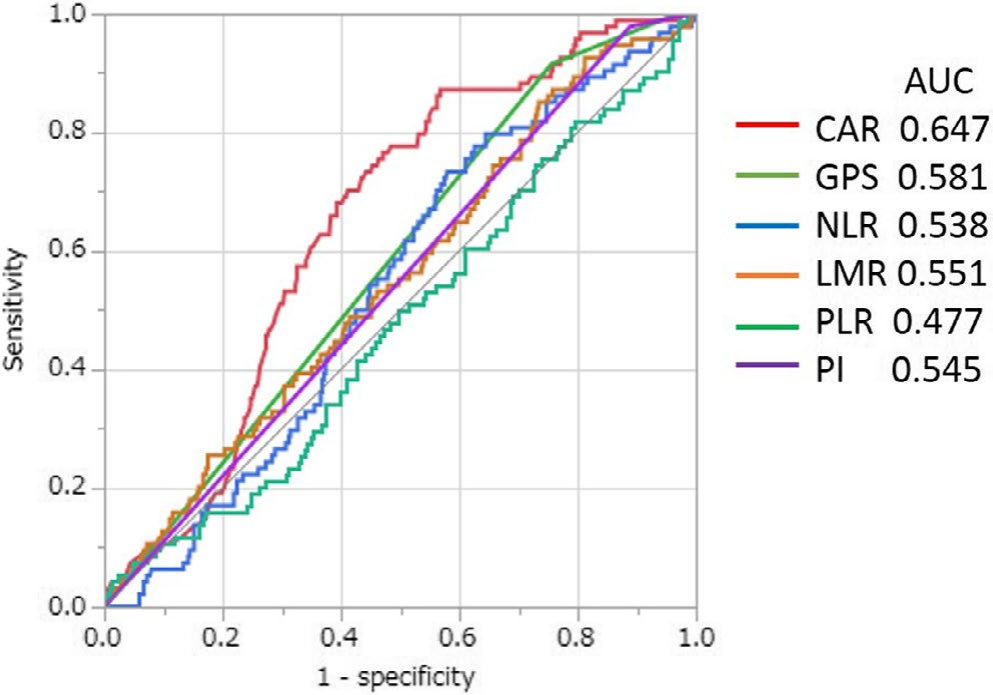
Comparison of the areas under the receiver operating curves for outcome prediction among the five inflammation‐based markers

A total of 482 patients were included in the study. Optimal cut‐off value for inflammation‐based markers was determined using the area under the curve (AUC) of ROC curves: CAR 0.027, AUC 0.647; GPS 1, AUC 0.581; NLR 2.2, AUC 0.538; LMR 3.3, AUC 0.551; PLR 106, AUC 0.477; and PI 1, AUC 0.545. The value for CAR was the highest and statistically significant among the inflammation‐based markers (Figure 1).

In the univariate analysis, statistically significant prognostic factors for poor OS rate included CRP level >0.20 mg/dL, albumin level <3.5 g/dL, AST level >35 IU/L, ICGR15 level >15%, AFP level >10 ng/mL, DCP level >100 mAU/mL, Child‐Pugh grade B, number of tumors >1, tumor size >50 mm, operation time >300 minutes, blood loss >1000 mL, liver cirrhosis, MVI, histological grade well and moderately differentiated, IM, low PNI, high CAR, high GPS, high NLR, and high PI. Multivariate analysis identified four indicators of poor OS (tumor size >50 mm, blood loss >1000 mL, liver cirrhosis and high CAR; Table 2). In the univariate analysis, statistically significant prognostic factors for poor disease‐free survival (DFS) rate included CRP level >0.20 mg/dL, albumin level <3.5 g/dL, AST level >35 IU/L, platelet count <14 × 10^4^/mm^3^, ICGR15 level >15%, AFP level >10 ng/mL, Child‐Pugh grade B, liver damage grades B and C, number of tumors >1, tumor size >50 mm, liver cirrhosis, MVI, IM, low PNI, high CAR, high GPS, high NLR, low LMR and high PI. Multivariate analysis identified six indicators of poor DFS (platelet count <14 × 10^4^/mm^3^, ICGR15 level >15%, number of tumors >1, MVI, high CAR and high GPS; Table 3). Among all the inflammation‐based markers, high CAR was the only factor negatively influencing both OS and DFS.

**Table 2:** Univariate and multivariate analyses of prognostic factors for overall survival

Variable	Univariate analysis			Multivariate analysis		
	*P* value	HR	95% CI	*P* value	HR	95% CI
Age, y (≤70/>70)	.942	1.015	0.656‐1.564			
Gender (male/female)	.665	0.893	0.551‐1.516			
BMI, kg/m^2^ (≤22/>22)	.882	1.033	0.661‐1.597			
HBV (N/Y)	.459	1.252	0.705‐2.431			
HCV (N/Y)	.899	1.028	0.665‐1.582			
CRP, mg/dL (≤0.20/>0.20)	<.001	2.996	1.915‐4.636	.563	1.256	0.579‐2.762
Alb, g/dL (≥3.5/<3.5)	<.001	2.519	1.525‐4.017	.353	2.038	0.432‐8.869
AST, IU/L (≤35/>35)	.028	1.619	1.051‐2.501	.871	1.048	0.592‐1.856
ALT, IU/L (≤34/>34)	.592	1.128	0.721‐1.743			
Plt, ×10^4^/mm^3^ (≥14/<14)	.099	1.437	0.933‐2.232			
PT, % (≥80/<80)	.838	0.948	0.552‐1.551			
T. Bil, mg/dL (≤1/>1)	.189	1.449	0.822‐2.413			
ICGR15, % (≤15/>15)	.037	1.584	1.026‐2.455	.343	1.331	0.734‐2.412
AFP, ng/mL (≤10/>10)	.022	1.665	1.074‐2.608	.244	1.368	0.807‐2.352
DCP, mAU/mL (≤100/>100)	<.001	2.356	1.525‐3.675	.212	1.492	0.794‐2.805
Child‐Pugh (A/B)	.012	2.378	1.224‐4.225	.608	1.246	0.524‐2.835
Liver damage (A/B, C)	.078	1.528	0.951‐2.395			
Tumor number (1/>1)	<.001	2.666	1.731‐4.135	.277	1.403	0.757‐2.574
Tumor size, mm (≤50/>50)	<.001	3.068	1.935‐4.775	.042	2.089	1.024‐4.236
Anatomical resection (Y/N)	.274	1.305	0.814‐2.172			
Operation time, min (≤300/>300)	.001	2.109	1.331‐3.439	.934	1.026	0.546‐1.964
Blood loss, mL (≤1000/>1000)	<.001	2.988	1.667‐5.045	.037	2.211	1.051‐4.427
LC (N/Y)	.041	1.631	1.018‐2.571	.013	2.227	1.179‐4.278
MVI (N/Y)	<.001	3.193	2.031‐4.945	.103	1.625	0.904‐2.886
Histological grade						
Well, moderately differentiated/poorly differentiated	<.001	10.58	2.351‐186.7	.799	0.404	0.321‐0.857
IM (N/Y)	<.001	4.579	2.825‐7.234	.127	1.765	0.847‐3.602
PNI (≥40/<40)	<.001	2.958	1.915‐4.567	.377	0.707	0.338‐1.548
CAR (≤0.027/>0.027)	<.001	3.976	2.556‐6.275	.046	2.171	1.012‐4.462
GPS (0/1, 2)	<.001	2.982	1.876‐4.656	.602	1.537	0.292‐7.658
NLR (≤2.2/>2.2)	.028	1.629	1.052‐2.511	.773	1.098	0.571‐2.062
LMR (≥3.3/<3.3)	.081	1.541	0.947‐2.433			
PLR (≤106/>106)	.788	1.061	0.688‐1.648			
PI (0/1, 2)	.015	2.231	1.177‐3.901	.361	0.558	0.137‐1.886

AFP, alpha‐fetoprotein; Alb, albumin; ALT, alanine aminotransferase; AST, aspartate aminotransferase; BMI, body mass index; CAR, C‐reactive protein to albumin ratio; CRP, C‐reactive protein; DCP, des‐gamma‐carboxyprothrombin; GPS, Glasgow prognostic score; HBV, hepatitis B virus; HCV, hepatitis C virus; ICGR15, indocyanine green retention rate at 15 min; IM, intrahepatic metastasis; LC, liver cirrhosis; LMR, lymphocyte to monocyte ratio; MVI, microvascular invasion; NLR, neutrophil to lymphocyte ratio; PI, prognostic index; PLR, platelet to lymphocyte ratio; Plt, platelet count; PNI, prognostic nutritional index; PT, prothrombin time; T. Bil, total bilirubin.

**Table 3:** Univariate and multivariate analyses of prognostic factors for disease‐free survival

Variable	Univariate analysis			Multivariate analysis		
	*P* value	HR	95% CI	*P* value	HR	95% CI
Age, y (≤70/>70)	.806	1.033	0.795‐1.339			
Gender (male/female)	.231	1.212	0.887‐1.689			
BMI, kg/m^2^ (≤22/>22)	.174	0.833	0.641‐1.085			
HBV (N/Y)	.723	1.065	0.756‐1.546			
HCV (N/Y)	.242	1.168	0.901‐1.521			
CRP, mg/dL (≤0.20/>0.20)	<.001	2.048	1.529‐2.715	.905	1.033	0.603‐1.774
Alb, g/dL (≥3.5/<3.5)	<.001	2.361	1.691‐3.235	.324	0.637	0.244‐1.544
AST, IU/L (≤35/>35)	.002	1.515	1.166‐1.966	.341	1.162	0.852‐1.583
ALT, IU/L (≤34/>34)	.129	1.229	0.941‐1.599			
Plt, ×10^4^/mm^3^ (≥14/<14)	.046	1.302	1.003‐1.694	.022	1.471	1.056‐2.058
PT, % (≥80/<80)	.499	1.108	0.817‐1.483			
T. Bil, mg/dL (≤1/>1)	.747	1.059	0.736‐1.482			
ICGR15, % (≤15/>15)	<.001	1.684	1.296‐2.191	.027	1.501	1.046‐2.144
AFP, ng/mL (≤10/>10)	.003	1.472	1.132‐1.916	.058	1.335	0.989‐1.805
DCP, mAU/mL (≤100/>100)	.052	1.302	0.997‐1.696			
Child‐Pugh (A/B)	.018	1.748	1.104‐2.632	.853	0.947	0.525‐1.653
Liver damage (A/B, C)	<.001	1.647	1.239‐2.171	.208	0.771	0.509‐1.155
Tumor number (1/>1)	<.001	2.228	1.713‐2.893	<.001	1.766	1.271‐2.441
Tumor size, mm (≤50/>50)	<.001	1.774	1.297‐2.389	.477	1.166	0.757‐1.763
Anatomical resection (Y/N)	.092	1.268	0.961‐1.661			
Operation time, min (≤300/>300)	.116	1.233	0.949‐1.608			
Blood loss, mL (≤1000/>1000)	.241	1.306	0.825‐1.966			
LC (N/Y)	.011	1.457	1.091‐1.929	.249	1.222	0.867‐1.714
MVI (N/Y)	<.001	2.101	1.555‐2.802	.002	1.797	1.231‐2.591
Histological grade						
Well, moderately differentiated/poorly differentiated	.282	1.264	0.832‐2.023			
IM (N/Y)	<.001	2.235	1.537‐3.159	.264	1.323	0.804‐2.128
PNI (≥40/<40)	<.001	2.211	1.682‐2.888	.089	0.705	0.477‐1.056
CAR (≤0.027/>0.027)	<.001	2.181	1.671‐2.841	.012	1.813	1.145‐2.789
GPS (0/1, 2)	<.001	3.173	2.361‐4.229	.031	2.791	1.102‐7.597
NLR (≤2.2/>2.2)	.046	1.296	1.003‐1.684	.525	1.126	0.777‐1.617
LMR (≥3.3/<3.3)	.011	1.473	1.097‐1.954	.342	1.213	0.811‐1.796
PLR (≤106/>106)	.257	1.163	0.894‐1.508			
PI (0/1, 2)	<.001	2.647	1.799‐3.778	.208	0.572	0.221‐1.347

AFP, alpha‐fetoprotein; Alb, albumin; ALT, alanine aminotransferase; AST, aspartate aminotransferase; BMI, body mass index; CAR, C‐reactive protein to albumin ratio; CRP, C‐reactive protein; DCP, des‐gamma‐carboxyprothrombin; GPS, Glasgow prognostic score; HBV, hepatitis B virus; HCV, hepatitis C virus; ICGR15, indocyanine green retention rate at 15 min; IM, intrahepatic metastasis; LC, liver cirrhosis; LMR, lymphocyte to monocyte ratio; MVI, microvascular invasion; NLR, neutrophil to lymphocyte ratio; PI, prognostic index; PLR, platelet to lymphocyte ratio; Plt, platelet count; PNI, prognostic nutritional index; PT, prothrombin time; T. Bil, total bilirubin.

**Figure 2:**
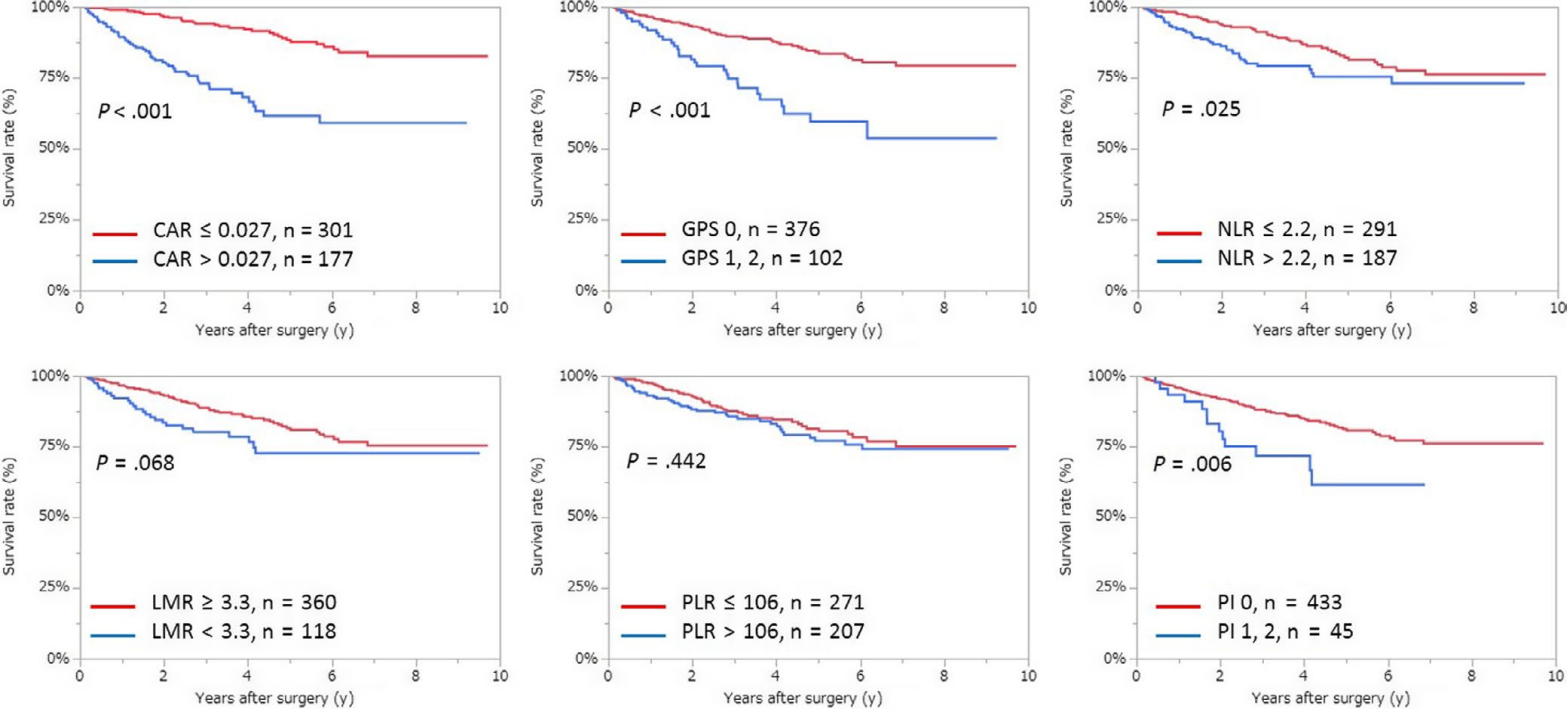
Relationship between the two groups of overall survival of different inflammation‐based markers. CAR, C‐reactive protein to albumin ratio; GPS, Glasgow prognostic score; LMR, lymphocyte to monocyte ratio; NLR, neutrophil to lymphocyte ratio; PI, prognostic index; PLR, platelet to lymphocyte ratio

**Figure 3:**
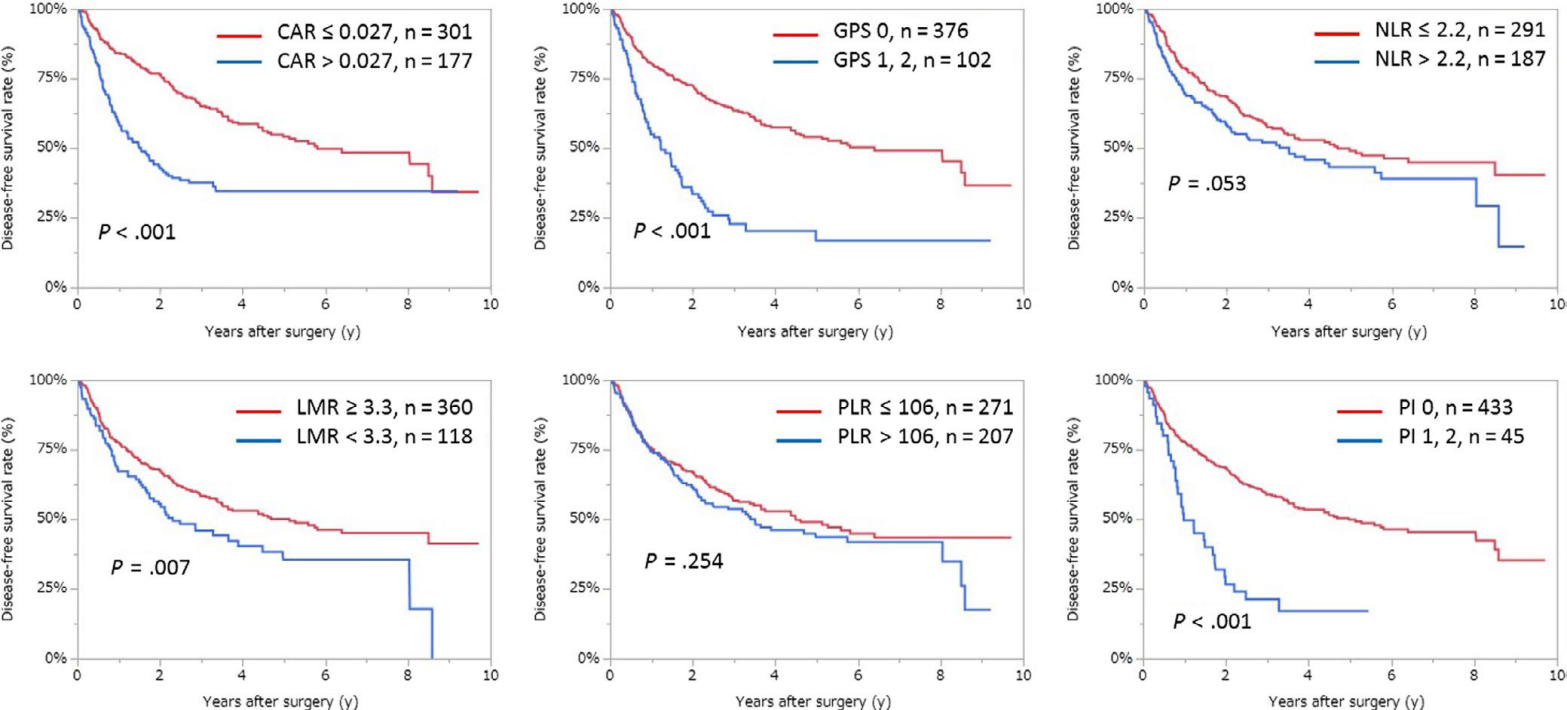
Relationship between the two groups of disease‐free survival of different inflammation‐based markers. CAR, C‐reactive protein to albumin ratio; GPS, Glasgow prognostic score; LMR, lymphocyte to monocyte ratio; NLR, neutrophil to lymphocyte ratio; PI, prognostic index; PLR, platelet to lymphocyte ratio

Kaplan‐Meier analyses showing OS and DFS using inflammation‐based markers are shown in Figures 2 and 3. As shown in Figure 2, Kaplan‐Meier analyses indicated that CAR, GPS, NLR and PI were correlated with OS, whereas LMR and PLR were not. In addition, Figure 3 shows that CAR, GPS, LMR, and PI were correlated with DFS, but NLR and PLR were not.

All baseline clinical characteristics were compared between the low and high CAR groups. Before propensity score matching, baseline parameters of the low and high CAR groups statistically differed in gender, BMI, HBV, HCV, platelet count, DCP, Child‐Pugh grade, liver damage, tumor size, operation time, blood loss and MVI (Table 4). After propensity score matching, all baseline clinical characteristics between the low and high CAR groups were well balanced (Table 4).

**Table 4:** Clinicopathological characteristics of the patients before and after propensity score matching

Variable	Before matching			After matching		
	CAR ≤0.027 (N = 301)	CAR >0.027 (N = 177)	*P* value	CAR ≤0.027 (N = 124)	CAR >0.027 (N = 124)	*P* value
Age, y (≤70/>70)	161/140 (53.5/46.5)	92/85 (52/48)	.776	61/63 (49.2/50.8)	67/57 (54/46)	.525
Gender (male/female)	221/80 (73.4/26.6)	146/31 (82.5/17.5)	.025	98/26 (79/21)	98/26 (79/21)	1
BMI, kg/m^2^ (≤22/>22)	132/169 (43.8/56.2)	60/116 (34.1/65.9)	.042	41/83 (33/67)	48/76 (38.7/61.3)	.427
HBV (N/Y)	243/56 (81.3/18.7)	157/19 (89.2/10.8)	.026	109/15 (87.9/12.1)	108/16 (87.1/12.9)	1
HCV (N/Y)	120/180 (40/60)	102/75 (57.6/42.4)	<.001	62/62 (50/50)	61/63 (49.2/50.8)	1
Plt, ×10^4^/mm^3^ (≥14/<14)	123/178 (40.9/59.1)	113/64 (63.8/36.2)	<.001	65/59 (52.4/47.6)	70/54 (56.5/43.5)	.611
PT, % (≥80/<80)	229/71 (76.3/23.7)	123/54 (69.5/30.5)	.106	91/32 (74/26)	91/33 (73.4/26.6)	1
T‐Bil, mg/dL (≤1/>1)	254/47 (84.4/15.6)	142/35 (80.2/19.8)	.259	105/19 (84.7/15.3)	101/23 (81.5/18.5)	.611
AST, IU/L (≤35/>35)	181/119 (60.3/39.7)	93/84 (52.5/47.5)	.103	75/49 (60.5/39.5)	64/60 (51.6/48.4)	.201
ALT, IU/L (≤34/>34)	195/106 (64.8/35.2)	105/72 (59.3/40.7)	.241	87/37 (70.2/29.8)	77/47 (62.1/37.9)	.227
ICGR15, % (≤15/>15)	172/124 (58.1/41.9)	95/79 (54.6/45.4)	.499	62/62 (50/50)	66/58 (53.2/46.8)	.703
AFP, ng/mL (≤10/>10)	163/134 (54.9/45.1)	89/86 (50.9/49.1)	.444	77/47 (62.1/37.9)	66/57 (53.7/46.3)	.198
DCP, mAU/mL (≤100/>100)	205/92 (69/31)	86/90 (48.9/51.1)	<.001	77/47 (62.1/37.9)	73/51 (58.9/41.1)	.696
Child‐Pugh (A/B)	283/17 (94.3/5.7)	156/21 (88.1/11.9)	.021	113/11 (91.1/8.9)	113/11 (91.1/8.9)	1
Liver damage (A/B, C)	227/67 (77.2/22.8)	118/57 (67.4/32.6)	.023	85/39 (68.6/31.4)	88/36 (71/29)	.782
Tumor number (1/>1)	205/96 (68.1/31.9)	106/71 (59.9/40.1)	.074	86/38 (69.4/30.6)	75/49 (60.5/39.5)	.183
Tumor size, mm (≤50/>50)	272/29 (90.4/9.6)	115/61 (65.3/34.7)	<.001	102/22 (82.3/17.7)	102/22 (82.3/17.7)	1
Anatomical resection (Y/N)	98/203 (32.6/67.4)	44/132 (25/75)	.096	39/85 (31.5/68.5)	39/85 (31.5/68.5)	1
Operation time, min (≤300/>300)	147/150 (49.5/50.5)	64/112 (36.4/63.6)	.005	59/65 (47.6/52.4)	54/70 (43.6/56.4)	.611
Blood loss, mL (≤1000/>1000)	277/22 (92.6/7.4)	149/27 (84.7/15.3)	.007	114/10 (91.9/8.1)	112/12 (90.3/9.7)	.823
MVI (N/Y)	250/51 (83.1/16.9)	131/46 (74/26)	.018	98/26 (79/21)	93/31 (75/31)	.546
LC (N/Y)	209/78 (72.8/27.2)	116/45 (72.1/27.9)	.912	82/32 (71.9/28.1)	77/39 (66.4/33.6)	.393
Histological grade						
Well, moderately differentiated/poorly differentiated	271/22 (92.5/7.5)	135/30 (81.8/18.2)	<.001	99/17 (85.3/14.7)	105/9 (92.1/7.9)	.144
IM (N/Y)	242/41 (85.5/14.5)	153/12 (92.7/7.3)	.023	114/10 (91.9/8.1)	112/12 (90.3/9.7)	.823

AFP, alpha‐fetoprotein; ALT, alanine aminotransferase; AST, aspartate aminotransferase; BMI, body mass index; CAR, C‐reactive protein to albumin ratio; DCP, des‐gamma‐carboxyprothrombin; HBV, hepatitis B virus; HCV, hepatitis C virus; ICGR15, indocyanine green retention rate at 15 min; IM, intrahepatic metastasis; LC, liver cirrhosis; LMR, lymphocyte to monocyte ratio; MVI, microvascular invasion; Plt, platelet count; PT, prothrombin time; T‐Bil, total bilirubin.

**Figure 4:**
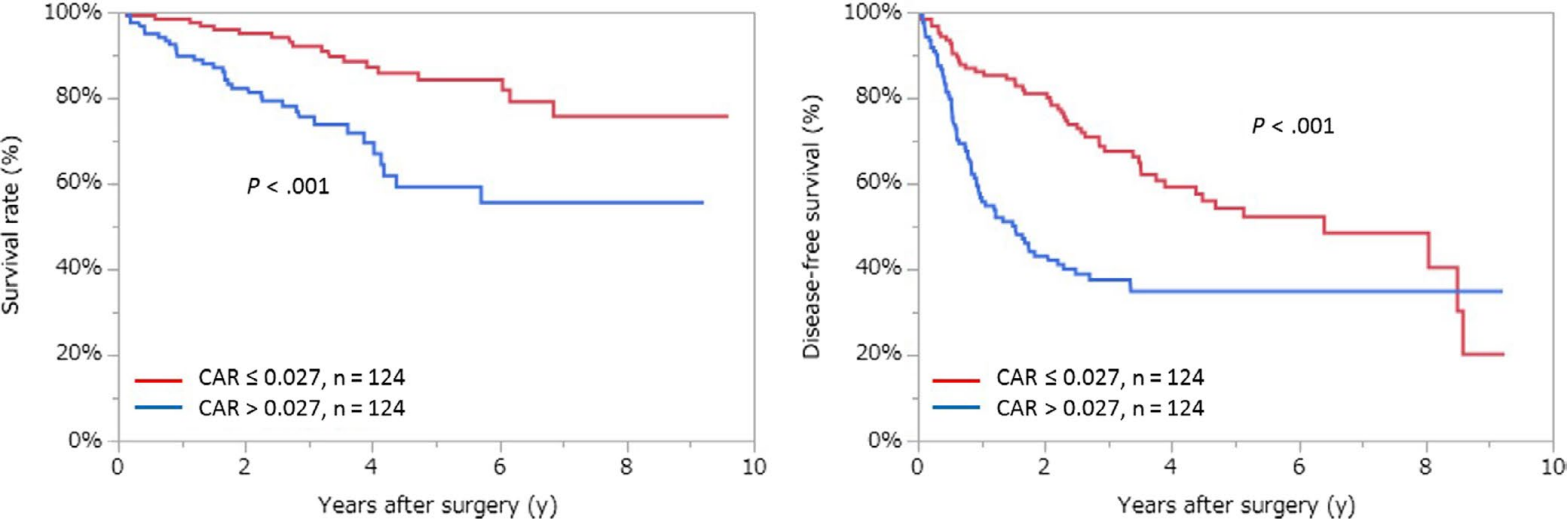
Relationship between the two groups of overall survival and disease‐free survival of CAR after propensity score matching. CAR, C‐reactive protein to albumin ratio

In total, 124 of the 301 patients with low CAR and an equal number of the 177 patients with high CAR were matched. As shown in Figure 4, Kaplan‐Meier analyses indicated that high CAR was correlated with poor OS and DFS.

## 4 DISCUSSION

The present study showed that high CAR, tumor size, blood loss and liver cirrhosis were correlated with poor OS, and that platelet count, ICGR15, multiple tumors, MVI, high CAR, and high GPS were correlated with poor DFS in multivariate analysis. For an evaluation of preoperative patient prognosis, only CAR was regarded as an independent prognostic factor for both OS and DFS in various inflammation‐based prognostic markers. Furthermore, the differences between the high and low CAR groups in clinicopathological characteristics such as gender, BMI, HBV, HCV, platelet count, DCP, Child‐Pugh grade, liver damage, tumor size, operation time, blood loss, MVI, histological grade, and IM were reduced by using propensity score matching (Table 4). On the basis of this background, high CAR was significantly associated with OS and DFS. Even after confounding factors were excluded by propensity score matching, CAR could be used to stratify patients into different risk groups.

Usefulness of CAR in predicting the prognosis of HCC has been reported previously.^11^, ^18^, ^19^, ^20^ The present study included only cases in which hepatectomy was carried out for HCC and the diagnosis of HCC was confirmed pathologically. Hence, this study probably had the largest cohort among the papers already published.^11^, ^20^ In addition, the results from the propensity score matching can enhance the usefulness of CAR. Similarity of the cut‐off values between our study and those reported by Shimizu et al,^11^ who have also examined cases of hepatectomy in confirmed HCC, could explain the difference in CAR cut‐off values between our study and previous reports^18^, ^19^ as being due to the variety of treatments that HCC patients have received.

Development of various inflammatory indices was due to the importance of the host inflammatory response in predicting clinical outcomes of patients with HCC. Previous studies detected elevated CRP levels in patients with carcinoma and showed that CRP levels were closely associated with HCC.^21^, ^22^ CRP is an acute‐phase reactant produced by hepatocytes and regulated by inflammatory cytokines, particularly interleukin (IL)‐6.^23^ Elevated IL‐6 and CRP levels are known to be associated with a higher risk of HCC.^24^ Therefore, CRP level plays a key role in the progression of HCC.^11^ With a high CAR, serum albumin level is found to be decreased. Hypoalbuminemia not only reflects liver dysfunction as a result of the underlying chronic liver disease, but is also associated with a sustained systemic inflammatory response, either from the tumor itself or as a host reaction. Circulating catabolic factors, such as tumor necrosis factor‐α and interleukins, mediate the hypoalbuminemia process.^25^ As albumin decreases along with the disease status, weight and muscle mass decrease, leading to a decrease in performance status and an increase in mortality.^26^

Among the inflammation‐based markers, lymphocytes reflect the host immune system's ability to recognize and eliminate tumors.^27^ Neutrophils can prompt the secretion of vascular endothelial growth factor (VEGF) and induce tumor growth.^28^ Monocytes infiltrate the stroma of tumors, accelerating tumor proliferation, angiogenesis and metastasis. Platelets promote tumor growth and angiogenesis through increased levels of VEGF and angiogenesis‐regulating chemokines.^29^ This contributes to the development of inflammation‐based markers. Results of the present study show that CAR and GPS scores, which were based on CRP, an acute‐phase protein, were superior in terms of differentiating patients with good prognosis from those with poor prognosis compared to those based on components of circulating white cell count (NLR, LMR, and PLR), as reported in a previous study.^30^

C‐reactive protein to albumin ratio and GPS were independent factors of DFS; CAR, but not GPS, was also an independent factor of OS. Most HCC patients have chronic hepatitis. Both serum albumin and CRP levels are decreased in HCC patients compared with their levels in patients with other types of cancer because albumin and CRP production are decreased in HCC patients with chronic hepatitis or liver cirrhosis.^23^ Albumin and CRP were decreased most apparently in patients with a GPS of 2 who have the worst postoperative survival among patients with the three types of GPS, because the cut‐offs for both serum albumin and CRP levels are used in the calculation of GPS. In contrast, as CAR is the ratio of CRP to albumin, it does not affect postoperative prognostic evaluation. Therefore, CAR predicts patient outcome more precisely than GPS as a result of the presence of continuous variables.

There are a few limitations in the present study. First, it was a retrospective study, although propensity score matching was used. As such, selection bias and potential confounders could not be avoided completely. Second, it was a single‐center study. Prospective cohort studies in multiple institutions should be carried out to confirm these results.

In conclusion, our study showed that CAR was the most useful prognostic indicator among inflammation‐based markers for patients who had undergone hepatectomy for HCC.

## DISCLOSURE

Conflicts of Interest: Authors declare no conflicts of interest for this article.

## References

[1] Global, regional, and national age‐sex specific all‐cause and cause‐specific mortality for 240 causes of death, 1990–2013: a systematic analysis for the Global Burden of Disease Study 2013. Lancet. 2015;385(9963):117–71. 25530442 10.1016/S0140-6736(14)61682-2 PMC4340604

[2] Bertuccio P , Turati F , Carioli G , Rodriguez T , La Vecchia C , Malvezzi M , et al. Global trends and predictions in hepatocellular carcinoma mortality. J Hepatol. 2017;67(2):302–9. 28336466 10.1016/j.jhep.2017.03.011

[3] Zaydfudim VM , Vachharajani N , Klintmalm GB , Jarnagin WR , Hemming AW , Doyle MB , et al. Liver resection and transplantation for patients with hepatocellular carcinoma beyond Milan criteria. Ann Surg. 2016;264(4):650–8. 27433910 10.1097/SLA.0000000000001866 PMC5279918

[4] Poon RT . Prevention of recurrence after resection of hepatocellular carcinoma: a daunting challenge. Hepatology (Baltimore, MD). 2011;54(3):757–9. 10.1002/hep.24569 21793027

[5] Pinato DJ , Sharma R , Allara E , Yen C , Arizumi T , Kubota K , et al. The ALBI grade provides objective hepatic reserve estimation across each BCLC stage of hepatocellular carcinoma. J Hepatol. 2017;66(2):338–46. 27677714 10.1016/j.jhep.2016.09.008

[6] Liu PH , Hsu CY , Hsia CY , Lee YH , Su CW , Huang YH , et al. Prognosis of hepatocellular carcinoma: assessment of eleven staging systems. J Hepatol. 2016;64(3):601–8. 26551516 10.1016/j.jhep.2015.10.029

[7] Diakos CI , Charles KA , McMillan DC , Clarke SJ . Cancer‐related inflammation and treatment effectiveness. Lancet Oncol. 2014;15(11):e493–503. 25281468 10.1016/S1470-2045(14)70263-3

[8] Mantovani A , Allavena P , Sica A , Balkwill F . Cancer‐related inflammation. Nature. 2008;454(7203):436–44. 18650914 10.1038/nature07205

[9] Chen L , Zhang Q , Chang W , Du Y , Zhang H , Cao G . Viral and host inflammation‐related factors that can predict the prognosis of hepatocellular carcinoma. Eur J Cancer. 2012;48(13):1977–87. 22325840 10.1016/j.ejca.2012.01.015

[10] Hanahan D , Weinberg RA . Hallmarks of cancer: the next generation. Cell. 2011;144(5):646–74. 21376230 10.1016/j.cell.2011.02.013

[11] Shimizu T , Ishizuka M , Suzuki T , Tanaka G , Shiraki T , Sakuraoka Y , et al. The value of the C‐reactive protein‐to‐albumin ratio is useful for predicting survival of patients with Child‐Pugh Class A undergoing liver resection for hepatocellular carcinoma. World J Surg. 2018;42(7):2218–26. 29288307 10.1007/s00268-017-4446-0

[12] Abe T , Tashiro H , Kobayashi T , Hattori M , Kuroda S , Ohdan H . Glasgow prognostic score and prognosis after hepatectomy for hepatocellular carcinoma. World J Surg. 2017;41(7):1860–70. 28197709 10.1007/s00268-017-3909-7

[13] Lai Q , Castro Santa E , Rico Juri JM , Pinheiro RS , Lerut J . Neutrophil and platelet‐to‐lymphocyte ratio as new predictors of dropout and recurrence after liver transplantation for hepatocellular cancer. Transpl Int. 2014;27(1):32–41. 24118272 10.1111/tri.12191

[14] Yang T , Zhu J , Zhao L , Mai K , Ye J , Huang S , et al. Lymphocyte to monocyte ratio and neutrophil to lymphocyte ratio are superior inflammation‐based predictors of recurrence in patients with hepatocellular carcinoma after hepatic resection. J Surg Oncol. 2017;115(6):718–28. 28127774 10.1002/jso.24549

[15] Peng W , Li C , Zhu WJ , Wen TF , Yan LN , Li B , et al. Prognostic value of the platelet to lymphocyte ratio change in liver cancer. J Surg Res. 2015;194(2):464–70. 25577142 10.1016/j.jss.2014.12.021

[16] Yamamura K , Sugimoto H , Kanda M , Yamada S , Nomoto S , Nakayama G , et al. Comparison of inflammation‐based prognostic scores as predictors of tumor recurrence in patients with hepatocellular carcinoma after curative resection. J Hepatobiliary Pancreat Sci. 2014;21(9):682–8. 24823966 10.1002/jhbp.114

[17] Kudo M , Kitano M , Sakurai T , Nishida N . General Rules for the Clinical and Pathological Study of Primary Liver Cancer, Nationwide Follow‐Up Survey and Clinical Practice Guidelines: The Outstanding Achievements of the Liver Cancer Study Group of Japan. Dig Dis. 2015;33(6):765–70. 26488173 10.1159/000439101

[18] Kinoshita A , Onoda H , Imai N , Iwaku A , Oishi M , Tanaka K , et al. The C‐reactive protein/albumin ratio, a novel inflammation‐based prognostic score, predicts outcomes in patients with hepatocellular carcinoma. Ann Surg Oncol. 2015;22(3):803–10. 25190127 10.1245/s10434-014-4048-0

[19] Chen J , Fang A , Chen M , Tuoheti Y , Zhou Z , Xu L , et al. A novel inflammation‐based nomogram system to predict survival of patients with hepatocellular carcinoma. Cancer Med. 2018;7(10):5027–35. 30259688 10.1002/cam4.1787 PMC6198220

[20] Ren Y , Fan X , Chen G , Zhou D , Lin H , Cai X . Preoperative C‐reactive protein/albumin ratio to predict mortality and recurrence of patients after curative resection with hepatocellular carcinoma. Med Clin. 2018, pii: S0025-7753(18)30727-9 10.1016/j.medcli.2018.11.010 30606506

[21] Zhao J , Liu J , Pang X , Wang S , Wu D , Zhang X , et al. Angiotensin II induces C‐reactive protein expression via AT1‐ROS‐MAPK‐NF‐kappaB signal pathway in hepatocytes. Cell Physiol Biochem. 2013;32(3):569–80. 24021937 10.1159/000354461

[22] She S , Jiang L , Zhang Z , Yang M , Hu H , Hu P , et al. Identification of the C‐reactive protein interaction network using a bioinformatics approach provides insights into the molecular pathogenesis of hepatocellular carcinoma. Cell Physiol Biochem. 2018;48(2):741–52. 30025407 10.1159/000491903

[23] Morris‐Stiff G , Gomez D , Prasad KR . C‐reactive protein in liver cancer surgery. Eur J Surg Oncol. 2008;34(7):727–9. 18356004 10.1016/j.ejso.2008.01.016

[24] Aleksandrova K , Boeing H , Nothlings U , Jenab M , Fedirko V , Kaaks R , et al. Inflammatory and metabolic biomarkers and risk of liver and biliary tract cancer. Hepatology (Baltimore, MD). 2014;60(3):858–71. 10.1002/hep.27016 PMC4231978 24443059

[25] Esper DH , Harb WA . The cancer cachexia syndrome: a review of metabolic and clinical manifestations. Nutr Clin Pract. 2005;20(4):369–76. 16207677 10.1177/0115426505020004369

[26] Roxburgh CS , McMillan DC . Cancer and systemic inflammation: treat the tumour and treat the host. Br J Cancer. 2014;110(6):1409–12. 24548867 10.1038/bjc.2014.90 PMC3960633

[27] Stotz M , Pichler M , Absenger G , Szkandera J , Arminger F , Schaberl‐Moser R , et al. The preoperative lymphocyte to monocyte ratio predicts clinical outcome in patients with stage III colon cancer. Br J Cancer. 2014;110(2):435–40. 24357796 10.1038/bjc.2013.785 PMC3899781

[28] Kuang DM , Zhao Q , Wu Y , Peng C , Wang J , Xu Z , et al. Peritumoral neutrophils link inflammatory response to disease progression by fostering angiogenesis in hepatocellular carcinoma. J Hepatol. 2011;54(5):948–55. 21145847 10.1016/j.jhep.2010.08.041

[29] Bambace NM , Holmes CE . The platelet contribution to cancer progression. J Thromb Haemost. 2011;9(2):237–49. 21040448 10.1111/j.1538-7836.2010.04131.x

[30] Proctor MJ , Morrison DS , Talwar D , Balmer SM , Fletcher CD , O'Reilly DS , et al. A comparison of inflammation‐based prognostic scores in patients with cancer. A Glasgow inflammation outcome study. Eur J Cancer. 2011;47(17):2633–41. 21724383 10.1016/j.ejca.2011.03.028

